# 2702. A Case Series of Kidney Transplant Recipients on Belatacept with CMV Infection in a Single Academic Center

**DOI:** 10.1093/ofid/ofad500.2313

**Published:** 2023-11-27

**Authors:** Fionna Feller, Elizabeth Cohen, Jennifer Marvin, Sarthak Virmani, Maricar F Malinis

**Affiliations:** Vanderbilt University Medical Center, Brentwood, Tennessee; Yale New Haven Hospital, New Haven, Connecticut; Yale New Haven Hospital, New Haven, Connecticut; Yale School of Medicine, New Haven, Connecticut; Yale University, New Haven, CT

## Abstract

**Background:**

*Cytomegalovirus* (CMV) infection has been increasingly observed among kidney transplant recipients (KTR) on belatacept for immunosuppression. Clinical outcomes of CMV infection among KTR on belatacept are not well described. We aimed to evaluate further in this case series from a single center.

**Methods:**

We performed a retrospective study of KTR on belatacept who developed CMV infection and were hospitalized between the period of 1/1/2017-12/31/2022. Data collected were: demographics, transplant and CMV parameters, allograft outcomes, and co-infection. Descriptive statistics was performed.

**Results:**

Of the 1,468 KTR, 387 received belatacept and have been hospitalized. Ten of the 387 (2.6%) had CMV infection. Among the 10, 60% were female and 20% were black. Median age was 59 years (range 32-78) and median time from transplant to first CMV infection was 6 months (range 1-228). Five had CMV DNAemia and 5 had CMV disease. Among the 5 with CMV DNAemia, 1 was CMV D+/R- and 4 was CMV R+. Of the 5 with CMV disease, 2 were CMV D+/R- and 3 were CMV R+. Corresponding clinical outcomes are reported in **Figure 1**.

Belatacept administration was uninterrupted in 7/10 KTR. Of these 7, the highest median peak and the longest duration of CMV DNAemia were seen in the CMV D+/R- only. CMV infection relapse (3/7), allograft rejection (1/7), and allograft loss (1/7) occurred in those with uninterrupted belatacept infusion. Bacterial co-infections occurred in 6/10 KTR. All survived > 90 days after CMV diagnosis. KTR with interrupted belatacept (n=3) were eventually changed to alternative regimen of azathioprine, tacrolimus, and everolimus.

**Table 1.**
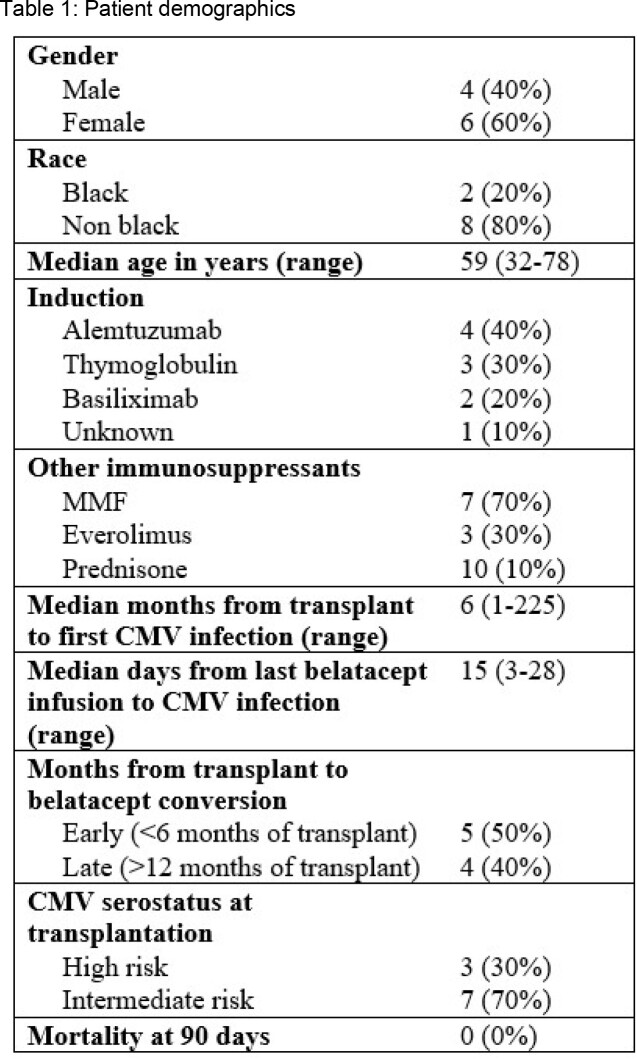
Patient demographics

**Figure 1.**
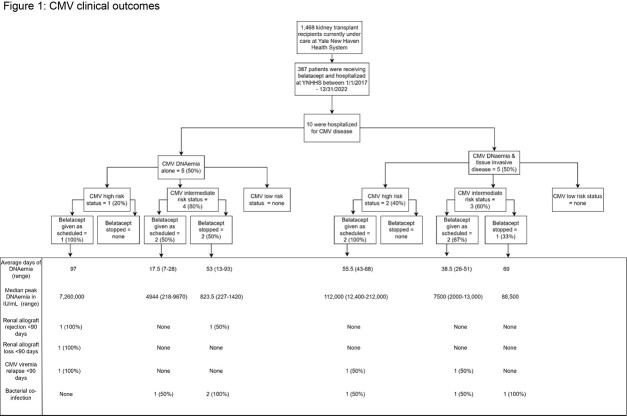
CMV clinical outcomes

**Conclusion:**

High risk KTR who received uninterrupted belatacept at time of CMV infection were observed to have the high level and prolonged duration of CMV DNAemia. Renal allograft rejection/loss and CMV relapse were also observed with uninterrupted belatacept administration regardless of CMV donor serostatus. This limited data suggests that KTR with either CMV D+/R- or CMV R+ may have poor clinical outcomes with continued belatacept administration in the setting of CMV infection. Prospective, multi-center studies are needed to better assess the impact of belatacept management during CMV infection.

**Disclosures:**

**All Authors**: No reported disclosures

